# Reactive oxygen species- and nitric oxide-dependent regulation of ion and metal homeostasis in plants

**DOI:** 10.1093/jxb/erad349

**Published:** 2023-09-05

**Authors:** Luisa M Sandalio, Jesús Espinosa, Sergey Shabala, José León, María C Romero-Puertas

**Affiliations:** Stress, Development and Signaling in Plants, Estación Experimental del Zaidín, Granada, Spain; Stress, Development and Signaling in Plants, Estación Experimental del Zaidín, Granada, Spain; School of Biological Science, University of Western Australia, Crawley, WA 6009, Australia; International Research Centre for Environmental Membrane Biology, Foshan University, Foshan, China; Institute of Plant Molecular and Cellular Biology (CSIC-UPV), Valencia, Spain; Stress, Development and Signaling in Plants, Estación Experimental del Zaidín, Granada, Spain; Universidad de Sevilla, Spain

**Keywords:** Heavy metals, ion channels, nutrients, post-translational regulation, reactive nitrogen species, reactive oxygen species, signaling, transporters, transcriptional regulation

## Abstract

Deterioration and impoverishment of soil, caused by environmental pollution and climate change, result in reduced crop productivity. To adapt to hostile soils, plants have developed a complex network of factors involved in stress sensing, signal transduction, and adaptive responses. The chemical properties of reactive oxygen species (ROS) and reactive nitrogen species (RNS) allow them to participate in integrating the perception of external signals by fine-tuning protein redox regulation and signal transduction, triggering specific gene expression. Here, we update and summarize progress in understanding the mechanistic basis of ROS and RNS production at the subcellular level in plants and their role in the regulation of ion channels/transporters at both transcriptional and post-translational levels. We have also carried out an *in silico* analysis of different redox-dependent modifications of ion channels/transporters and identified cysteine and tyrosine targets of nitric oxide in metal transporters. Further, we summarize possible ROS- and RNS-dependent sensors involved in metal stress sensing, such as kinases and phosphatases, as well as some ROS/RNS-regulated transcription factors that could be involved in metal homeostasis. Understanding ROS- and RNS-dependent signaling events is crucial to designing new strategies to fortify crops and improve plant tolerance of nutritional imbalance and metal toxicity.

## Teaser

ROS and RNS are involved in nutrient and heavy metal stress sensing, signaling, and adaptive responses. They act as important hubs in the regulation of expression levels and functionality of ion channels/transporters.

## Introduction

Currently, more than a billion people suffer from malnourishment, while a similar number lack basic micronutrients, such as Zn, Fe, and Cu, in their diet. These deficiencies have a major impact on human health, and forecasts predict that this impact will become worse in the future (Semba *et al.*, 2022). At the same time, one of the consequences of anthropogenic action is the accumulation of heavy metals, which are very harmful to all types of organisms (Huang *et al.*, 2020). Soil pollution has become a major issue worldwide, with an increase of polluted areas in China, Australia, the USA, and Europe in particular (Yang *et al.*, 2022). Some of these contaminated farmlands are still used to cultivate crops, posing a high risk to human health (Yang *et al.*, 2022).

Another major contaminant in the soil is high amounts of salts (predominantly NaCl), which accumulate as a result of either natural causes (e.g. rock weathering) or inappropriate agricultural practices such as the use of low-quality water for irrigation. Globally, the area of soil affected by salinity is increasing at an alarming rate of 2–3 ha min^–1^ (Shabala *et al.*, 2014), and soil salinity is expected to affect over 50% of the world population in the future (Liu *et al.*, 2020). Salinity stress tolerance was present in the ancestors of crops but has been significantly weakened or lost during domestication (Palmgren *et al.*, 2015; Lopez-Marques *et al.*, 2020; Chen *et al.*, 2021). As a result, all major staple crops (rice, wheat, maize) are highly sensitive to soil salinity.

An efficient way to deal with heavy metal contamination and salinity would be to develop crops that are able to take up significant quantities of heavy metals or salts from the soil without a yield penalty and, at the same time, prevent their accumulation in the edible plant parts. To achieve this aim, a deep understanding of the mechanisms that regulate the uptake, translocation, and sequestration of salt and heavy metals is required.

When plants are exposed to hostile soil conditions (e.g. nutritional deficiencies, salinity, or the presence of heavy metals), they increase their production of reactive oxygen species (ROS) such as **·**OH, H_2_O_2_, O_2_·^–^, and ^1^O_2_. These ROS originate as by-products of aerobic metabolism and their accumulation is determined by the balance between their production and their elimination by antioxidant systems (Sandalio *et al.*, 2012; Halliwell and Gutteridge, 2015). Uncontrolled levels of ROS are toxic: they cause oxidative stress and result in damage to various macromolecules (lipids, proteins, and DNA). However, ROS (mainly H_2_O_2_) also have an important signaling role in the control of processes such as growth, development, or the response to different biotic and abiotic stress conditions (Sandalio *et al*., 2012; Peláez-Vico *et al.*, 2022). Transcriptomic studies have shown the existence of specificity in ROS signaling and responses induced by different stimuli (Gadjev *et al*., 2006; Vaahtera *et al*., 2014). The mechanisms involved are not well known but require the intervention of Ca^2+^ signals and other molecules such as nitric oxide (NO) and various hormones (Peláez-Vico *et al.*, 2022; Shikha *et al*., 2022).

In the past years, an important role of ROS and NO in the regulation of ion channels and transporters of macro- and micronutrients as well as heavy metals has emerged (Zepeda-Jazo *et al*., 2011; Hafsi *et al.*, 2022), involving post-translational and transcriptional regulation, as well as hormone balance (Cui *et al*., 2018; Nieves-Cordones, *et al*., 2019; Shikha *et al*., 2022). However, the molecular mechanisms responsible for this regulation remain elusive. In this review, we summarize the current standing in the field and discuss the progress made in understanding the mechanistic basis of ROS and RNS production and their role in the regulation of ion transporters and channels, at both the transcriptional and post-translational levels. This information could be of interest in designing new strategies to develop fortified crops, improve plant tolerance of salinity, and devise new phytoremediation methodologies based on redox biochemistry governed by ROS and RNS.

## Production of reactive oxygen and nitrogen species at the subcellular level

### Reactive oxygen species production and metabolism

The term ROS includes reduced oxygen species such as H_2_O_2_, radicals such as **·**OH and O_2_**·**^–^, and excited forms of oxygen, the singlet oxygen ^1^O_2_ (Halliwell and Gutteridge, 2015; Sandalio *et al.*, 2021). The chemical reactivity and biological functions of ROS differ considerably: H_2_O_2_ is the most stable form, which can even move between organelles and cells through aquaporins (Smirnoff and Arnaud, 2019; Peláez-Vico *et al.*, 2022), whereas **·**OH is the most reactive and short-lived of all ROS (Demidchik, 2014). ROS occur as a normal attribute of aerobic life, and their production and removal needs to be balanced by specific antioxidant defenses (Halliwell and Gutteridge, 2015). ROS such as **·**OH can be very reactive and oxidize almost all kinds of molecules, including proteins, lipids, and DNA, promoting oxidative damage that can even give rise to cell death (Halliwell and Gutteridge, 2015; Sandalio *et al.*, 2023). This situation can be triggered by changes in the environment that alter ROS homeostasis, such as nutritional disturbances, drought, salinity, high or low temperatures, or the presence of different pollutants (Nieves-Cordones *et al*., 2019; Cejudo *et al.*, 2021). Cells have developed complex mechanisms to detect and regulate these changes to maintain metabolic functionality. ROS are also used as secondary messengers, operating in the detection of environmental changes and triggering specific changes at the transcriptional and post-translational levels (Mhamdi and Van Breusegem, 2018; Sandalio *et al.*, 2019; Romero-Puertas *et al.*, 2022).

**Fig. 1.**
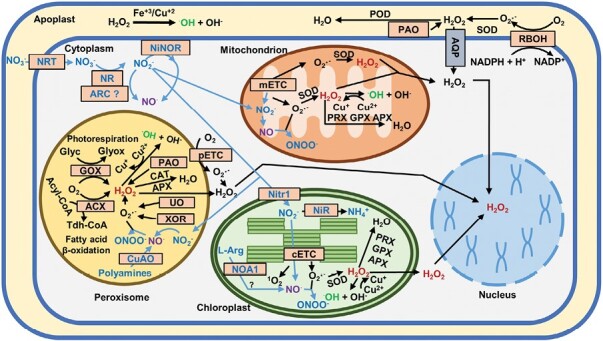
Reactive oxygen and nitrogen species metabolism at the subcellular level in plant cells. ACX, acyl-CoA oxidase; APX, ascorbate peroxidase; AQP, aquaporin; ARC, amidoxime reducing component; CAT, catalase; CuAO, copper-containing amine oxidases; ETC, electron transport chain (p, peroxisomal; c, chloroplastidial; m, mitochondrial); Glyc, glycolate; Glyox, glyoxylate; GOX, glycolate oxidase; GPX, glutathione peroxidase; NiNOR, nitrite:NO-reductase; NiR, nitrite reductase; Nitr1, nitrite transporter; NOA1, NO-associated 1 protein; NR, nitrate reductase; NRT, nitrate transporter; PAO, polyamine oxidase; POD, peroxidase; PRX, peroxiredoxin; RBOH, respiratory burst oxidase homolog; SOD, superoxide dismutase; Tdh-CoA, *trans*-2,3-dehydroacyl-CoA; UO, urate oxidase; XOR, xanthine oxidoreductase.

ROS production and redox compartmentalization in organelles is an effective evolutionary strategy to regulate physiological process and the cellular response to stress conditions through site-specific ROS footprinting (Jones and Go, 2010; Romero-Puertas *et al.*, 2022). The subcellular redox network facilitates rapid responses to changes in the intracellular redox equilibrium, which, in turn, regulates signaling processes and cell responses (Sandalio *et al.*, 2021; Zoccarato *et al.*, 2022). ROS production takes place in different cell organelles, such as chloroplasts, mitochondria, and peroxisomes, as a consequence of electron transport chains in these organelles, with the production of O_2_·^–^ and further dismutation to H_2_O_2_ (Smirnoff and Arnaud, 2019; Phua *et al.*, 2021; Sandalio *et al.*, 2021) (Fig. 1). However, ROS production is also associated with metabolic pathways such as photorespiration (glycolate oxidase, GOX), polyamine metabolism (polyamine oxidases, PAO; copper amine oxidase, CuAO), catabolism of ureides (xanthine oxidoreductase, XOR; urate oxidase, UO), and β-oxidation of fatty acids in peroxisomes (Acyl-CoA oxidase, ACX) (Sandalio *et al*., 2021) (Fig. 1). In the apoplast, ROS are mainly produced in the plasma membrane by the NADPH oxidases (also called respiratory burst oxidase homologs, RBOHs; Pei *et al*., 2000; Peláez-Vico *et al.*, 2022) and peroxidases (Lüthje and Martinez-Cortes, 2018; Smirnoff and Arnaud, 2019). Singlet oxygen is mainly produced in chloroplasts (Dogra and Kim, 2020). **·**OH is produced by Fenton-type reactions requiring the participation of O_2_**·**^–^, H_2_O_2_, and Fe or Cu, and therefore could be produced in any organelle (Demidchik, 2014; Halliwell and Gutteridge, 2015). ROS levels in plants are tightly regulated by a range of enzymatic and non-enzymatic antioxidants (Smirnoff and Arnaud, 2019; Phua *et al*., 2021; Sandalio *et al*., 2021) (Fig. 1).

**Fig. 2.**
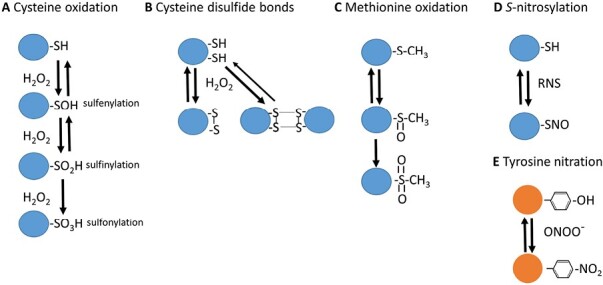
ROS- and RNS-dependent PTMs. Some of the most relevant redox-dependent PTMs associated with ROS and RNS are shown. These modifications include (A) cysteine oxidation (sulfenylation, sulfinylation, and sulfonylation), (B) cysteine disulphide bonds, (C) methionine oxidation, (D) cysteine *S*-nitrosylation, and (E) tyrosine nitration.

Signaling by H_2_O_2_ occurs through the reversible oxidation of specific cysteine residues from proteins to sulfenic acid (Young *et al*., 2019; Sies *et al.*, 2022). Owing to their transient nature, these sulfur modifications are considered as redox switches (Young *et al.*, 2019). Redox post-translational modification (PTM) of proteins, such as methionine oxidation, sulfenylation, and sulfinylation, as well as intra- and inter-molecular disulfide bond formation, are rapid and reversible mechanisms that regulate protein function in living cells in response to changing redox states (Fig. 2) (Sandalio *et al*., 2019; Young *et al.*, 2019), while other modifications, such as sulfonylation and carbonylation, give rise to irreversible oxidation, inactivation, and further degradation of proteins (Sandalio *et al.*, 2023). The reversible modifications can fine-tune protein function, localization, stability, and interactions in response to redox changes, to adapt the cell to environmental changes and mitigate potential damage (Young *et al.*, 2019). Protein PTMs also trigger cell signaling pathways and cross-talk among complex interconnected signaling pathways by affecting protein–protein interactions that underpin plant stress responses. These signaling events are coupled with ROS-activated MAP kinase cascades and transcriptional regulation in both plant and animal cells (Li *et al.*, 2022; Sies *et al.*, 2022).

### Nitric oxide production and metabolism

The way NO is synthesized and sensed in plants as well as the signaling mechanisms underlying its regulatory functions is complex and remains controversial (León and Costa-Broseta, 2020). NO can be synthesized from either oxidized or reduced N-containing precursors, with nitrate being the most abundant and relevant source that is reduced by the cytosolic nitrate reductases (NRs) through nitrite as an intermediate (Yamasaki and Sakihama, 2000; Rockel *et al.*, 2002; León and Costa-Broseta, 2020) (Fig. 1). Other molybdoenzymes, such as amidoxime reducing component (ARC) in the cytoplasm in *Chlamydomonas* (Chamizo-Ampudia *et al.*, 2016), xanthine oxidoreductases/dehydrogenases (XORs/XDHs) in the cytoplasm (Cantu-Medellin and Kelley, 2013), and peroxisomes (Sandalio *et al.*, 2021, 2023), can also catalyze the biosynthesis of NO from nitrite. Other oxidized sources, such as xanthine, can be used under specific conditions in different photosynthetic organisms (Godber *et al.*, 2000; Maia and Moura, 2015) (Fig. 1). Although an oxidative pathway involving NOS has also been proposed to be operational in plants (Kolbert *et al*., 2019), no NOS orthologs have been identified in plants (Jeandroz *et al.*, 2016). Peroxisomes have, however, been reported to be a source of NOS-like activity in plants (Sandalio *et al.*, 2023), although this needs further experimental support and the identification of the enzymes involved. Other oxidative pathways from polyamines or hydroxylamine, such as CuAOs, localized in the apoplast and peroxisomes have been proposed as alternative sources of NO in plants (Tun *et al.*, 2006; Rümer *et al.*, 2009; Wimalasekera *et al.*, 2011; Planas-Portell *et al.*, 2013; Zhou *et al.*, 2016; Groß *et al.*, 2017; Sandalio *et al*., 2023) (Fig. 1). The only NOS identified in the marine green alga *Ostreococcus taurii* (Foresi *et al.*, 2010) was predicted to be a cytosolic enzyme (Gaudet *et al.*, 2011), but experimental support is still needed. Under conditions of limited oxygen availability, mitochondria are an important source for NO production, with nitrite being an efficient electron acceptor (Fig. 1) (Planchet *et al.*, 2005). The excess of NO produced in mitochondria in hypoxic conditions is modulated by the action of the phytoglobin–NO cycle (Igamberdiev *et al.*, 2005).

NO, as a free radical, tends to react with other molecules, such as ROS, although the resulting molecules, such as hydroxyl radicals (**·**OH), are centers of molecular damage (Nappi and Vass, 1998; Bright *et al.*, 2006; Sandalio *et al.*, 2019). Some of these reactions have a relevance for NO-triggered signaling, such as the reaction of NO with superoxide-yielding peroxynitrite (ONOO^–^) (Radi *et al.*, 2001; Arasimowicz-Jelonek and Floryszak-Wieczorek, 2011; Vandelle and Delledonne, 2011) (Fig.1), which is involved in the PTM of proteins by nitration of tyrosine residues (Lozano-Juste *et al.*, 2011; León, 2022). On the other hand, the metabolic reaction of NO with the redox regulator glutathione also has high relevance in NO-triggered signaling, as the resulting *S*-nitrosoglutathione (GSNO) (Fig.1) is an excellent NO donor in reactions of transnitrosylation, including the *S*-nitrosylation of the cysteine residues of proteins (Astier *et al.*, 2012; Zaffagnini *et al.*, 2016) (Fig. 2). The master regulator of protein *S*-nitrosylation is the enzyme *S*-nitrosoglutathione reductase (GSNOR) (Fig.1) (Jahnová *et al*., 2019), which regulates GSNO levels (Feechan *et al.*, 2005) and, at the same time, is regulated by the *S*-nitrosylation of key cysteine residues (Guerra *et al.*, 2016; Zhan *et al.*, 2018). Both NO-derived PTMs (Fig. 2) of proteins have a deep impact on the regulation of growth and development as well as responses to stress (Lindermayr *et al.*, 2005; Astier and Lindermayr, 2012; Romero-Puertas *et al.*, 2013; Sami *et al.*, 2018; Sandalio *et al.*, 2019). In fact, PTMs are the mechanism of action of NO known to date, and the recent identification of NO-regulated chromatin-modifying histone deacetylases and transcription factors may explain the role of NO in epigenetic mechanisms and modulation of gene transcription (Wurm and Lindermayr, 2021). Furthermore, NO-mediated PTMs control the redox state of cells by acting on the enzymes of the most prominent antioxidant systems and NO biosynthetic and metabolic enzymes (Pietraforte *et al.*, 2003; Romero-Puertas and Sandalio, 2016; Begara-Morales *et al.*, 2016; Sandalio *et al.*, 2019; Costa-Broseta *et al.*, 2021).

## Reactive oxygen- and nitrogen-dependent regulation of metal transporters and ion channels

The transport and accumulation of essential macronutrients (N, P, K, S, Ca, Mg) and micronutrients (e.g. Fe, Cu, Zn, Mn) is mediated by transporters and channels located at the plasma membrane and tonoplast. Some of these are highly selective for specific ions whereas others may allow the passage of a large number of ions. In addition, some of them may share transcription factors or certain essential components of the signaling network that regulates their expression (Ohkama-Ohtsu and Wasaki, 2010). The above scenario is also applicable to non-essential heavy metals. For example, cadmium (Cd) does not use specific transporters and accumulates in plants through transporters of other elements, such as Fe (IRT1, NRAMP) or Zn (ZIP, ZRT), among others (Tao and Lu, 2022). ROS seem to play an important role in the root response to nutrient deprivation (Nieves-Cordones *et al.*, 2019) and excess of heavy metals (Hafsi *et al.*, 2022). Furthermore, changes in the plant transcriptome in NO-related mutants or in response to NO donors also involve the transport category, where the ATP-binding cassette (ABC) transporter family is usually well represented (Parani *et al.*, 2004; Besson-Bard *et al.*, 2009; Gibbs *et al.*, 2014; Hussain *et al.*, 2016) (Table 1). Only a few reports regarding the regulation of metal transporters by NO- and ROS-dependent PTMs are available, and information regarding their functionality is even scarcer. A search in the Plant PTM Viewer database (https://www.psb.ugent.be/webtools/ptm-viewer/index.php) resulted in 1317 proteins in *Arabidopsis thaliana* being the target of methionine oxidation, of which only five were identified as transporters; 3438 proteins that are targeted for reversible cysteine oxidation, five of which were identified as transporters; and 6836 proteins that can potentially experience sulfenylation, of which only 11 were identified as transporters and only two were related to metal transporters (ABC19 and ABCF1/GCN1; Huang *et al.*, 2019). Among 1833 Arabidopsis proteins identified as possible targets of *S*-nitrosylation, only one of the three transporters identified in the *gsnor1-3* mutant is associated with metal transport (Hu *et al.*, 2015) (Table 1). To get an insight into the potential relevance of post-translational regulation by NO-derived PTMs of the metal transporters, we performed an *in silico* analysis of the potential nitration and *S*-nitrosylation of Arabidopsis metal transporters. We took advantage of the available tools for computational prediction of NO-dependent PTMs by using GPS-SNO (https://sno.biocuckoo.org/; Xue *et al.*, 2010) and GPS-YNO2 (https://yno2.biocuckoo.org/; Liu *et al.*, 2011) for the prediction of *S*-nitrosylation and tyrosine nitration sites, respectively, and DeepNitro (http://deepnitro.renlab.org; Xie *et al.*, 2018), which allows both predictions simultaneously. We analyzed 91 Arabidopsis metal transporters belonging to the ABC, COPT, FRO, HMA, IRT, MRS2, NRAMP, OPT, POT, YSL, and ZIP families. Supplementary Table S1 summarizes the predicted NO-dependent PTMs with either *S*-nitrosylation or tyrosine nitration sites, although their functionality needs to be tested in direct experiments. Among the transporters predicted to be modified by both tools, ABCB25 is potentially *S*-nitrosylated at C^639^, HMA3A and HMA3B at C^384^, HMA5 at C^10^, POT5 at C^762^, and ZIP8 at C^41^. We also identified with high confidence tyrosine nitration sites for several transporters, including ABCC6 at Y^695^, ABCG36 at Y^933^, ABCG40 at Y^891^, HMA2 at Y^10^, MRS2-1 at Y^259^, and the high-affinity potassium transporter POT5 at Y^783^. Other transporters, such as the vacuolar Fe exporters NRAMP3 and NRAMP4, were identified as nitrated in our *in silico* analysis by only one of the prediction tools. Unfortunately, there is no information available on the functional role of the tyrosine residues that are potentially modified, so the relevance of these predictions should be experimentally confirmed and their functional implications analyzed.

**Table 1.** Regulation of metal and ion transporters by ROS and NO

Channel/transporter	Regulation by ROS		Regulation by NO		References
	TR	PTM	TR	PTM	
AtNRAMP3	⇧⇩		⇧⇩		Gagnot *et al.* (2008); Farinati *et al.* (2010); Molins *et al.* (2013); Gibbs *et al.* (2014); Hafsi *et al.* (2022); Shee *et al.* (2022)
AtNRAMP4			⇧		Shee *et al.* (2022)
OsNRAMP5			⇧		Singh *et al.* (2016); A.P. Singh *et al*. (2017)
AtNRAMP6	⇩				Hafsi *et al.* (2022)
AtIRT1	⇧⇩		⇧		Bahmani *et al.* (2019); Hafsi *et al.* (2022)
LeIRT1			⇧		Graziano and Lamattina (2007); Jin *et al.* (2009); Liu *et al.* (2022)
OsIRT1			⇧		Singh *et al.* (2016); A.P. Singh *et al*. (2017)
OsIRT2				✓	Yang *et al.* (2016)
AtIRT3			⇩		Shanmugam *et al.* (2011)
AtZIP5			⇩		Gibbs *et al.* (2014)
AtZIP9			⇧		Gibbs *et al.* (2014)
AtZIP11			⇩		Gibbs *et al.* (2014)
AtABCC2			⇩		Gibbs *et al.* (2014)
AtABCC3	⇧				Farinati *et al.* (2010)
TaABCC3	⇧				Bhati *et al.* (2015)
AtABCC4			⇩		Gibbs *et al.* (2014)
TaABCC4	⇧				Bhati *et al.* (2015)
AtABCC6	⇧				Terrón-Camero *et al.* (2022)
TaABCC6	⇧				Bhati *et al.* (2015)
AtABCC8			⇩		Gibbs *et al.* (2014)
TaABCC9	⇩				Bhati *et al.* (2015)
AtABCC10			⇩		Gibbs *et al.* (2014)
TaABCC13	⇧				Bhati *et al.* (2015)
AtABCC14			⇧		Gibbs *et al.* (2014)
TaABCC14	⇩				Bhati *et al.* (2015)
TaABCC16	⇩				Bhati *et al.* (2015)
AtABCG36	⇧⇩		⇩	✓	Hu *et al.* (2015); Jalmi *et al.* (2018); Bahmani *et al.* (2019); Sheng *et al.* (2019); Li *et al.* (2022)
AtABCG40	⇩				Terrón-Camero *et al.* (2022)
AtCAX1			⇩		Gibbs *et al.* (2014)
AtCAX3	⇩		⇩		Gibbs *et al.* (2014); Bahmani *et al.* (2019)
Ccc1 (VIT1 ortholog)		✓			Li *et al.* (2010); Sorribes-Dauden *et al.* (2020)
AtVIT2		✓			Jacques *et al.* (2015)
AtVTL1		✓	⇧		Gibbs *et al.* (2014); Jacques *et al.* (2015)
AtVTL2			⇧		Gibbs *et al.* (2014)
AtCOPT5		✓			Liu *et al* (2014)
OsOPT3				✓	Yang *et al.* (2016)
AtHMA4	⇧				Farinati *et al.* (2010)
AtHMA6				✓	Hu *et al.* (2015)
AtHMA7			⇧		Gibbs *et al.* (2014)
AtMTP1			⇧		Gibbs *et al.* (2014)
AtEIN2			⇧		Gibbs *et al.* (2014)
AT1G29820			⇧		Gibbs *et al.* (2014)
AT5G23760			⇩		Gibbs *et al.* (2014)
AtGORK	⇧⇩	✓			Demidchik *et al.* (2003, 2010); Tran *et al.* (2013); Shabala *et al.* (2016); Wang *et al.* (2017); Hafsi *et al.* (2022)
PsGORK		✓			Zepeda-Jazo *et al.* (2011)
HvGORK		✓			Velarde-Buendía *et al.* (2012)
AtSKOR		✓			Garcia-Mata *et al*. (2010)
AtKUP5			⇧		Gibbs *et al.* (2014)
AtKUP6			⇩		Gibbs *et al.* (2014)
AtKUP8	⇧				Hafsi *et al.* (2022)
AtHAK5	⇧		⇩		Gibbs *et al.* (2014); Hafsi *et al.* 2022); Kim *et al.* 2010); Wang *et al.* (2021)
AtAKT1			⇩		Xia *et al.* (2014)
AtAKT2			⇧		Gibbs *et al.* (2014)
AtKT2			⇧		Gibbs *et al.* (2014)
AtNHX2			⇧		Gibbs *et al.* (2014)
AtKEA1			⇧		Gibbs *et al.* (2014)
BnCNGC1			⇩		Huang *et al.* (2022)
AtPmitoKATP		✓		✓	Chiandussi *et al.* (2002); Pastore *et al.* (2007)
AtTPC1		✓			Pottosin *et al.* (2009)
AtANN1		✓			Laohavisit *et al*. (2012)
Ca^2+^ channels		✓			Pei *et al.* (2000); Demidchik *et al.* (2003, 2007); Evans *et al.* (2005); Demidchik and Maathuis (2007); Breygina *et al.* (2016)
NSCCs		✓			Pei *et al.* (2000); Demidchik *et al*. (2003, 2010, 2018); Zepeda-Jazo *et al.* (2011); Velarde- Buendía *et al.* (2012)
AtHIP06			⇩		Gibbs *et al.* (2014)
AtHIP13			⇩⇩		Gibbs *et al.* (2014)
AtHIPP20			⇧⇧		Gibbs *et al.* (2014)
AtHIPP21			⇧		Gibbs *et al.* (2014)
AtHIPP22			⇧		Gibbs *et al.* (2014)
AtHIPP32			⇧		Gibbs *et al.* (2014)
AtHIPP34		✓			Liu *et al.* (2014)
AtHIPP35			⇧		Gibbs *et al.* (2014)
AtHIPP39			⇩		Gibbs *et al.* (2014)
AtHIPP43			⇧		Gibbs *et al.* (2014)

PTM, post-translational modification; TR, transcriptional regulation. Up and down arrows indicate up- and down-regulation, respectively. ✓ indicates PTM by ROS or NO.

### Natural resistance-associated macrophage proteins

**Fig. 3.**
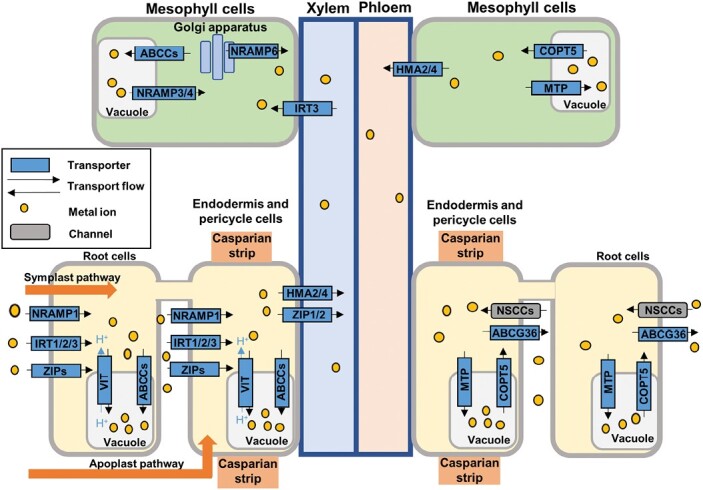
Scheme of the uptake, accumulation, and translocation of metals in plants. Metals are absorbed by the roots through the apoplastic and symplastic pathways. They are transported into the cytosol by different transporters associated with the plasma membrane and can be further transported to the vacuole through tonoplast-associated transporters. In addition, metals are translocated to mesophyll cells through the xylem, where can they accumulate in the vacuole.

Natural resistance-associated macrophage proteins (NRAMPs) are an evolutionarily conserved family of proteins that function as proton-coupled metal ion transporters that can transport Mn^2+^, Fe^2+^, Zn^2+^, Cu^2+^, Cd^2+^, Al^3+^, Co^2+^, and Ni^2+^ in prokaryotic and eukaryotic organisms (Nevo and Nelson, 2006; Banerjee and Datta, 2020; Yang *et al.*, 2022). In Arabidopsis, six genes encode members of the NRAMP transporter family (NRAMP1–NRAMP6). AtNRAMP1 is located in the plasma membrane of root cells (Fig. 3) and functions as a high-affinity Mn^2+^ transporter under Mn deficiency (Cailliatte *et al.*, 2010). AtNRAMP2 is a Mn^2+^ transporter localized in the *trans*-Golgi network; knock down of *AtNRAMP2* promotes a reduction of cellular redox homeostasis under Mn deficiency (Alejandro *et al.*, 2017; Gao *et al.*, 2018). AtNRAMP3 and AtNRAMP4 are functionally redundant and are involved in the release of metals from vacuoles; they are important for the retrieval of Fe^2+^ stores in seeds during germination, for the supply of Mn^2+^ to photosystem II in leaves, and for the response to Cd^2+^ stress (Thomine *et al.*, 2003; Lanquar *et al.*, 2004; Lanquar *et al.*, 2010; Molins *et al.*, 2013). AtNRAMP6 is localized to the Golgi/*trans*-Golgi network (Fig. 3), like AtNRAMP2, and plays an important role in intracellular Fe^2+^ homeostasis (Li *et al.*, 2019) and Cd^2+^ distribution within the cell (Tao and Lu, 2022). Several pieces of evidence demonstrate that ROS can regulate the expression and content of some NRAMPs in Arabidopsis plants. Molins *et al.* (2013) showed that AtNRAMP3 protein levels were increased in roots when plants were grown in media supplemented with Cd^2+^ and 1 mM H_2_O_2_ for 1 week, in agreement with a survey of the publicly available microarray data indicating that *AtNRAMP3* gene expression is up-regulated upon oxidative stress induced by H_2_O_2_, paraquat, and Fe excess (Gagnot *et al.*, 2008). In fact, *nramp3nramp4* Arabidopsis double mutants showed a hypersensitive phenotype when growing on media supplemented with 0.5 mM H_2_O_2_, suggesting that these transporters may be regulated by H_2_O_2_ (Molins *et al.*, 2013). Furthermore, Arabidopsis mutants lacking RBOH C, D and F (*rbohC*, *rbohD*, *rbohF*), the most important sources of ROS associated with the plasma membrane (Mittler, 2017), showed up-regulation of *AtNRAMP3* and *AtNRAMP6* after 24 h of 50 µM Cd^2+^ treatment, whereas no significant changes were observed in the wild-type (WT) genotype (Hafsi *et al.*, 2022). These results could explain the higher influx and accumulation of Cd observed in roots in response to 24 h of Cd treatment in *Atrboh* mutants, as well as the accumulation of Fe and Zn in roots, while translocation was inhibited for all three metals (Gupta *et al*., 2017; Hafsi *et al.*, 2022). These results indicate the existence of an RBOH-dependent H_2_O_2_ regulation of *AtNRAMP3* and *AtNRAMP6* expression under Cd stress conditions. In rice, *OsNRAMP5* has been shown to be induced by NO donors and modulated by NO in the plant response to arsenic (As) (Singh *et al.*, 2016; A.P. Singh *et al.*, 2017) (Table 1). Additionally, *NRAMP3* is up-regulated in the triple mutant *nia1nia2noa1-2* (Table 1), and NRAMP3 and NRAMP4 were predicted to be nitrated in our *in silico* analysis (Supplementary Table S1).

### ZIP family

The zinc regulated transporter and iron regulated transporter-like protein (ZIP) family (also known as ZRT, IRT-like protein) belongs to the IRT family (Yang *et al.*, 2022). The ZIP transporters mediate the uptake of Zn, Fe, and Mn into the cytosol from the extracellular space (Fig. 3). Zn deficiency up-regulates six ZIP family genes in *Hordeum vulgare* (Tiong *et al.*, 2015), and it has been suggested that ZIP transporters have an important role in plant adaptation to low and fluctuating Zn in soil in wheat (Niazkhani *et al*., 2021). Iron deficiency is one of the most important factors limiting crop production in the world, and IRT1 is the most important root transporter for the uptake of ferrous Fe from the soil (Zhang *et al*., 2019). IRT1 is highly expressed in Fe-deficient root cells to improve Fe absorption and therefore promote growth and development (Zhang *et al*., 2019). However, it can also mediate the uptake of other cations, such as Zn, Mn, Co, or Cd (Hu, 2021; Abuzeineh *et al.*, 2022; Assunção, 2022; Tao and Lu, 2022). Overexpression of *IRTI* in Arabidopsis and rice increased their sensitivity to Zn and Cd (reviewed in Tao and Lu, 2022). Bahmani *et al.* (2019) have reported the up-regulation of *AtIRT1* expression in Arabidopsis plants treated with H_2_O_2_ and NO donors for 24 h. In turn, the expression of the tobacco haemoglobin gene *NtHb1*, which acts as an NO scavenger, in Arabidopsis plants led to the down-regulation of *IRT1*, due to a reduction of NO and H_2_O_2_ in response to Cd exposure. Hafsi *et al.* (2022) showed differential expression of *IRT1* in Arabidopsis WT plants and *rboh* mutant plants deficient in RBOH C, D, and F after 24 h of 50 µM Cd treatment. This suggests the existence of an RBOH isoform-dependent H_2_O_2_ regulation of *IRT1* expression under Cd stress conditions. Other studies showed the NO-dependent induction of *LeIRT1* in tomato roots grown under Fe deficiency under normal and elevated CO_2_ levels (Graziano and Lamattina, 2007; Jin *et al.*, 2009), and in pear (Liu *et al.*, 2022). By contrast, a repression of *IRT1* in Arabidopsis roots under Cd stress to avoid Cd accumulation has been described (Connolly *et al.*, 2002, 2003; Hafsi *et al.*, 2022) (Table 1). *IRT1* repression was increased in the presence of a NOS-l inhibitor, also suggesting a role for NO in the regulation of *IRT1* under Cd stress (Besson-Bard *et al.*, 2009). Similar results were described in tomato roots grown under an excess of Cd, where the NO-dependent up-regulation of *IRT1* was responsible for nitrate-facilitated Cd accumulation in plants (Luo *et al.*, 2012). GSNO also induced Arabidopsis *IRT1* (García *et al.*, 2010). Transcriptomic analysis of the NO-deficient triple mutant *nia1nia2noa1-2* (Gibbs *et al.*, 2014), showed several transporters that were differentially up- or down-regulated compared with WT plants (Table 1). In accordance with previous results, *IRT1* is down-regulated in the NO-deficient mutant, while *IRT3* is up-regulated (Table 1), probably to compensate for *IRT1* down-regulation as overexpression of *IRT3* in *irt1-1* mutants recovers the *irt1-1* iron-deficient phenotype (Shanmugam *et al.*, 2011). Interestingly, *ZIP5* and *ZIP11* are up-regulated whereas *ZIP9* is down-regulated in the triple mutant *nia1nia2noa1-2* (Gibbs *et al.*, 2014) (Table 1). On the other hand, an iTRAQ-based proteomic analysis of plasma membrane-associated proteins in rice plants exposed to Cd stress after NO treatment allowed the identification of several differentially regulated metal transporters, including the Fe transporter IRT2 (Yang *et al.*, 2016). In addition, ZIP8, which is involved in metal transport in the rhizosphere and antioxidant activity (Wu *et al.*, 2016), is potentially *S*-nitrosylated at C^41^ based on our *in silico* analysis (Supplementary Table S1).

### Vacuolar iron transporters

The vacuolar iron transporter (VIT) family are tonoplast-localized transporters. They probably function as H^+^-dependent antiporters and are involved in fungi and plants in preventing the negative effects of Fe^2+^ excess (Yang *et al.*, 2022) (Fig. 3). In rice plants, OsVIT1 and OsVIT2 can transport Fe^2+^ and Zn^2+^ into the vacuole (Zhang *et al.*, 2012), while in wheat TaVIT2 may transport Fe^2+^ and Mn^2+^ (Connorton *et al.*, 2017). The Ca^2+^-sensitive cross-complementer 1 (Ccc1) is the VIT1 ortholog in yeast, and it has been suggested that ROS can increase the activity of Ccc1 transporter (Li *et al.*, 2010; Sorribes-Dauden *et al.*, 2020). Similarly, ROS increase the activity of the vacuolar Ccc1 transporter in fungi (Sorribes-Dauden *et al*., 2020), although the underlying mechanism has not been established in any organism. Therefore, similar regulation could be applied to the VIT proteins of plants. In fact, Arabidopsis VACUOLAR IRON TRANSPORTER1-LIKE1 (VTL1) has been identified as a target of methionine oxidation by a functional protein-bound methionine oxidation proteomic analysis using *Atcat2-2* mutants, and Arabidopsis VIT2 was identified as a target of oxidized cysteine that could be reversibly reduced (Jacques *et al.*, 2015). The *VIT1* gene, which is down-regulated under Fe deficiency in Arabidopsis, encodes a transporter involved in vacuolar Fe loading, while NRAMP4, a vacuolar Fe exporter, is up-regulated. Therefore, Fe homeostasis is regulated by different ROS- and NO-modulated transporters. Many examples of ROS and Fe signaling cross-talk have been observed in photosynthetic organisms, with ROS being an important signal to regulate Fe homeostasis and *vice versa* in plants (Thi Tuyet Le *et al.*, 2019). Together with NRAMPs, IRTs, and ZIPs, two VIT family proteins are also differentially expressed in the triple mutant *nia1nia2noa1-2* (Gibbs *et al.*, 2014) (Table 1). All these data suggest the existence of an NO-dependent fine-tuned Fe and Zn homeostasis mechanism to achieve optimum levels of these metals, probably not only in the triple mutant *nia1nia2noa1-2* but in general.

### Cu transporters

The COPT/Ctr Cu transporters play an important role in Cu^2+^ uptake and homeostasis, and are localized in the plasma membrane of root tip cells (Shin *et al.*, 2012). Arabidopsis COPT5 has been identified as a target of reversible cysteine oxidation by using OxiTRAQ, a quantitative redox proteomics approach (Liu *et al*., 2014), and therefore its activity could be regulated by ROS-dependent redox changes. Yang *et al.* (2016) also identified OPT3 as differentially regulated in an iTRAQ proteomic analysis of plasma membrane-associated proteins in rice plants exposed to Cd stress after NO treatment.

### Heavy metal transport/detoxification superfamily proteins

Heavy metal transport/detoxification superfamily proteins (HMPs) play key roles in heavy metal transport and detoxification in plant cells. HMPs are metalloproteins or metallochaperone-like proteins containing heavy metal-associated (HMA) domains with two cysteine residues that bind and transfer Cu, Cd, Co, Zn, and other heavy metal ions (Li *et al.*, 2020). Plant proteins containing HMA domains fall into several groups: HPPs (heavy metal-associated plant proteins), HIPPs (heavy metal-associated isoprenylated plant proteins), ATX1-like and P1B-ATPase (heavy metal ATPases; HMAs). HMAs have been widely studied in different species (Li *et al.*, 2020; He *et al.*, 2020) and are mainly located at the plasma membrane, where they are involved in long-distance transport of ion metals such as Cd, Zn, and/or Cu (Fig. 3). In Arabidopsis, *HMA5* is induced by high Cu levels and causes the efflux of excess Cu from the cytosol to the plasma membrane (Li *et al.*, 2020); however, different HMPs can differ in their metal specificity and the organ in which they function (Li *et al.*, 2020). Information on the regulation of HMPs by ROS is scarce; however, a transcriptomic analysis of Arabidopsis WT and *acx1* mutant plants (Romero-Puertas *et al.*, 2022) revealed ACX1-dependent differential regulation of *HMP43* and *HMP20*, thus suggesting a possible regulation by H_2_O_2_ produced during β-oxidation in the cell. Interestingly, the analysis on the Arabidopsis *gox2* mutant transcriptome (Terrón-Camero *et al.*, 2022) did not show any change in the expression of these genes, suggesting a differential regulation of HMPs depending on the ROS source. Less information is available on ROS-dependent PTMs of this group of proteins. HIPP34 has been identified as a target of reversible cysteine oxidation in a functional OxiTRAQ analysis of Arabidopsis cultured cells exposed to H_2_O_2_ (Liu *et al.*, 2014). However, the functionality of this PTM requires further analysis. Interestingly, genes encoding the HMPs and HIPPs superfamily proteins are highly represented among the down-regulated genes in the *nia1nia2noa1-2* triple mutant (Table 1). On the other hand, in our *in silico* analysis the Cd/Zn-transporting ATPases HMA3A and HMA3B were predicted to be *S*-nitrosylated in a cytoplasmic loop at C^384^, which is close to the D^397^ that seems to act as a binding site in the 4-aspartylphosphate intermediate. The probable Cu-transporting ATPase HMA5, which is involved in Cu detoxification in roots (Andrés-Colás *et al.*, 2006), was predicted to be *S*-nitrosylated at C^10^, which in the AlphaFold three-dimensional structure is far from any of the C^62^, C^65^, C^140^, and C^143^ residues involved in Cu binding. Only C^65^ was also predicted to be *S*-nitrosylated, but by only one of the predictive tools (Supplementary Table S1). In turn, the nitration of HMA5 at C^10^, and of HMA2 at Y^10^, was also predicted with high confidence (Supplementary Table S1). Additionally, among the 926 endogenous *S*-nitrosylated proteins identified in Arabidopsis, the chloroplastic Cu-transporting ATPase PAA1 (HMA6, Q9SZC9) was identified (Hu *et al.*, 2015).

### ABC transporters

ABC transporters are a multimeric family of proteins that use ATP to transport a variety of substances (including carbohydrates, ions, lipids, xenobiotics, antibiotics, heavy metals, and drugs) to and from cells, mainly into the vacuole (Do *et al.*, 2021). ABC transporters are classified into eight subfamilies based on their structure and function (Do *et al.*, 2021). Only C-type ABC transporters (ABCC; classically referred to as multidrug resistance-associated proteins, MRPs) are found on the tonoplast, where they play a role in plant metal tolerance, maintaining the transport of metals into vacuoles to prevent their harmful effects (Jogawat *et al.*, 2021) (Fig. 3). As an example, AtABCC3 takes part in transporting phytochelatin and its complexes with Cd (Brunetti *et al.*, 2015), as well as other metals such as Mn and Zn, to the vacuole (Jogawat *et al.*, 2021), and ABCC4 transports Cd from the cytoplasm to the vacuole in *Ophiopogon japonicus* (Zhao *et al.*, 2022). Moreover, *AtABCC6*, which is also involved in metal tolerance, is induced in Arabidopsis plants exposed to Cd treatment (Gaillard *et al.*, 2008). Some members of the ABC family can be transcriptionally regulated by ROS. The up-regulation of *AtABCC6* in response to Cd treatment has been found to be GOX2-dependent in *Atgox2* mutants (Terrón-Camero *et al.*, 2022), thus suggesting an H_2_O_2_-dependent regulation of this transporter associated with photorespiration (Table 1). In wheat seedlings exposed to 10 µM H_2_O_2_, Bhati *et al.* (2015) showed up-regulation of the expression of *TaABCC3*, *TaABCC4*, *TaABCC6*, and *TaABCC13*, while *TaABCC9*, *TaABCC14*, and *TaABCC16* were down-regulated, suggesting that these transporters can be differentially regulated by H_*2*_O_2._ ABCG is the largest ABC transporter subfamily in plants and plays a critical role in heavy metal tolerance. AtABCG36/AtPDR8 is located at the plasma membrane of root cells and plays a role as a Cd extrusion pump (Kim *et al.*, 2007). Bahmani *et al.* (2019) showed that *PDR8* expression increased in response to Cd but decreased in plants treated with H_2_O_2_ and NO, and the opposite results were obtained in Arabidopsis plants expressing tobacco *Hb1*, with reduced production of ROS and NO. Wu *et al.* (2019) proposed a model in which Cd stress inhibits the expression of mitochondrial *MMDH2 (malate dehydrogenase 2*), reducing ROS levels, in turn leading to increased expression of *ABCG36,* which finally reduces Cd accumulation. Furthermore, Arabidopsis AtABCG40/PDR12 is located at the plasma membrane and is strongly induced by Pb^2+^ treatment, and its overexpression leads to plants being more Pb^2+^ tolerant due to Pb^2+^ efflux (Lee *et al.*, 2005). Transcriptomic analysis of WT and *Atgox2* mutant Arabidopsis after Cd stress for 24 h (Terrón-Camero *et al.*, 2022) allowed the identification of GOX2-dependent differential expression of *AtABCG40*, *AtABCG16*, and *ABCB12*/*PGP12*, suggesting a possible regulation of these transporters by peroxisomal H_2_O_2_. ABCB1 activity in rat brain capillaries is apparently regulated by NO produced by inducible NO synthase in combination with protein kinase C (PKC), with NO reducing the activity, although the underlying mechanism is not well known (Crawford *et al.*, 2018). Interestingly, transporters from the ABC family have been shown to be modulated by NO in plant response to As (P.K. Singh *et al*., 2017), and regulation of Cd transport through the activation of ABC transporters was shown to be one of the major mechanisms involved in NO-dependent Cd detoxification in tall fescue after an integrated transcriptomic and metabolomic analysis (Zhu *et al.*, 2020). The mitochondrial ABC transporter B family member 25 (ABCB25), which seems to be essential for exporting Fe/S cluster precursors from mitochondria into the cytoplasm (Bernard *et al.*, 2009) and mediates glutathione-dependent resistance to heavy metals (Kim *et al.*, 2006), was predicted in our *in silico* analysis to be *S*-nitrosylated at C^639^, close to the C-terminus and not located in a transmembrane domain (Supplementary Table S1). As mentioned before, we also identified with high confidence tyrosine nitration sites for several ABC transporters, including ABCC6 at Y^695^, ABCG36 at Y^933^, and ABCG40 at Y^891^ (Supplementary Table S1). Further analysis would be required to stablish the role of these tyrosine nitration sites in the regulation of those ABC transporters.

### Cation/proton exchanger (CAX) family

The cation/proton exchanger (CAX) family comprises vacuole-localized transmembrane antiporters that use secondary active transport to exchange cations from the cytoplasm with protons, transporting cations to the vacuole to maintain ion homeostasis in guard cells (Pittman and Hirschi, 2016; Yang *et al.*, 2022). Some CAX isoforms have broad substrate specificity, providing the ability to transport trace metal ions such as Mn^2+^ and Cd^2+^. AtCAX3 has been shown to be involved in the efflux of Ca^2+^, Zn^2+^, and Cd^2+^ in Arabidopsis plants (Yang *et al.*, 2022). In halophytic plants, CAXs have been reported to play a role in salt tolerance (Pittman and Hirschi, 2016). Bahmani *et al.* (2019) showed that the transcription of *AtCAX3* was increased in response to Cd in Arabidopsis WT plants, whereas it was decreased in plants treated with H_2_O_2_ and NO for 24 h, and increased in *NtHb1*-expressing Arabidopsis plants, thus demonstrating a redox regulation of *CAX3* expression (Table 1). The expression of *NtHb1* in Arabidopsis regulates Cd transporter expression by decreasing NO and ROS levels, down-regulating *IRT1* and *PDR8*, while up-regulating *CAX3*, giving rise to a reduction in the Cd levels in roots and shoots (Bahmani *et al.*, 2019). Additionally, *CAX1* is among the genes differentially regulated in the NO-deficient triple mutant *nia1nia2noa1-2* (Table 1).

### Non-selective cation channels

Ca^2+^-permeable channels have been shown to be activated by H_2_O_2_ in many plant systems, such as root epidermal cells (Demidchik *et al.*, 2003, 2007), guard cells (Pei *et al.*, 2000), and pollen tubes (Breygina *et al.*, 2016). In intact plants, this activation leads to an increase in cytosolic free Ca^2+^, which occurs in a dose-dependent manner (Leshem *et al.*, 2007; Ma and Berkowitz, 2011). However, in patch-clamp experiments H_2_O_2_ did not activate whole-cell currents in protoplasts isolated from the Arabidopsis mature root epidermis (Demidchik *et al.*, 2007), suggesting that the above stimulatory effects of H_2_O_2_ on the rapid rise in cytosolic free Ca^2+^ may be indirect and mediated by **·**OH produced in the cell walls (Demidchik, 2015). In addition, H_2_O_2_-induced activation of Ca^2+^ currents was observed only when H_2_O_2_ was applied to the cytosolic side of the membrane (Demidchik and Maathuis, 2007), implying a need for its transport (through aquaporins) across the plasma membrane for *in planta* operation. In mitochondria from mammalian cardiac muscle, the Ca^2+^ release channels/ryanodine receptors (RyR2s), which are cation-selective channels that have a high ion conductance for both monovalent (K^+^) and divalent (Ca^2+^) cations, can be regulated by oxidation and *S*-nitrosylation (Meissner, 2004). Twenty-one cysteine residues per RyR2 subunit were reported to be in a reduced state and could be potential targets for redox modifications including *S*-nitrosylation and disulfide cross-linking (Nikolaienko *et al*., 2018). In mammalian tissues, SR/ER Ca^2+^-ATPase (SERCA) can also be modified by cysteine oxidation or tyrosine nitration, while plasma membrane Ca^2+^ ATPase (PMCA) is inhibited either by its direct oxidation or by methionine oxidation in its binding partner calmodulin (O-Uchi *et al*., 2014).

While the above reports referred to Ca^2+^-permeable ion channels, no specific Ca^2+^-selective channels have been reported so far in plants (Demidchik *et al.*, 2018), and Ca^2+^ uptake across cellular membranes is mediated by non-selective cation channels (NSCCs). These NSCCs are permeable to a wide range of cations, including essential macronutrients (K^+^, Ca^2+^, Mg^2+^, NH_4_^+^) and micronutrients (Zn^2+^, Fe^2+^) (Demidchik and Maathuis, 2007), as well as toxic nutrients such as Na^+^, Cd^2+^, or Al^3+^. The Arabidopsis genome contains 40 NSCCs in total, divided into two main families: cyclic nucleotide gated channels (CNGCs; 20 genes in Arabidopsis) and ionotropic glutamate receptors (GLRs; 20 genes) (Maathuis, 2006; Demidchik and Maathuis, 2007). In addition, tonoplast-based TPC (Two-Pore Cation) channels and several types of mechanosensitive channels, such as MSL (MscS-Like), MCA (Mid1-Complementing Activity), and OSCA (hyperosmolality-gated Ca^2+^-permeable) are also classified as NSCCs (Basu and Haswell, 2017; Liu *et al.*, 2018).

Activation of NSCCs has been reported for several types of plant systems, both for H_2_O_2_ and for ·OH. In root epidermis, ·OH activates NSCCs, triggering a simultaneous Ca^2+^ influx and K^+^ efflux (following the electrochemical gradient for these ions); this activation has been observed in both the mature root zone and the root apex of a large number of plants (Demidchik *et al*., 2003; Zepeda-Jazo *et al.*, 2011;  Velarde-Buendía *et al*., 2012). In patch-clamp experiments, ·OH-induced activation of Ca^2+^ influx and K^+^ efflux conductances was reported in Arabidopsis roots (Demidchik *et al.*, 2003), although the mechanism has not been elucidated. The extent of ·OH-induced activation of NSCCs has often been negatively correlated with abiotic stress tolerance in plants, specifically with plants’ ability to adapt to soil salinity (Bose *et al.*, 2014; Wang *et al.*, 2018; Liu *et al.*, 2019). This is hardly surprising, as NSCCs are also permeable to Na^+^ (Demidchik and Tester, 2002) and salinity-stress induced accumulation of ROS in root tissues may form a positive feedback loop exacerbating Na^+^ uptake into root epidermis. Exogenous H_2_O_2_ activates Ca^2+^-permeable NSCCs in protoplasts isolated from Arabidopsis guard cells (Pei *et al.*, 2000) and in the outside-out mode from the cytoplasmic side in root epidermis (Demidchik *et al.*, 2003, 2007).

NO is also able to modulate Na and K nutrition in plants under salinity stress, as the Na transporter CNGC1 appears to be regulated by NO in *Brassica napus* (Huang *et al.*, 2022). Moreover, the function of the AKT1 channel, via overproduction of an active form of vitamin B6 (pyridoxal 5ʹ-phosphate), is repressed by NO in Arabidopsis (Xia *et al.*, 2014) (Table 1). The sodium exchanger-encoding gene *NHX2* has also been identified as one of the genes that are differentially regulated in the NO-deficient triple mutant *nia1nia2noa1-2* (Table 1). NO also selectively regulates abscisic acid-dependent Ca^2+^-sensitive K^+^ and Cl^–^ channels of *Vicia faba* guard cells, inducing Ca^2+^ release from intracellular stocks (García-Mata *et al.*, 2003) (Table 1). Ciorba *et al* (1999), using ShC/B voltage-dependent K^+^ channels expressed in *Xenopus* oocytes as a model system, demonstrated that NO slows down the time course of K^+^ channel inactivation by oxidizing a critical methionine residue in the inactivation ball domain of the channel protein. Additionally, the channel protein was protected from methionine oxidation by methionine sulfoxide reductase and vitamin C (Ciorba *et al.*, 1999).

### K^*+*^*-*selective efflux channels

Shaker-type depolarization-activated outward-rectifying K^+^-efflux GORK channels are present in both guard cells (hence their name—Guard Cell Outward Rectifying K^+^ channel) and root epidermis, and are known to be activated by ·OH. Discovered first in Arabidopsis (Demidchik *et al.*, 2003, 2010), ·OH-activated GORK channels have since been reported in pea root epidermis (Zepeda-Jazo *et al.*, 2011) and in barley root cells, where their activation correlated with salt sensitivity (Velarde-Buendía *et al*., 2012); more recently, ROS-activated GORK channels were found to be essential for Arabidopsis responses to hypoxia stress (Wang *et al.*, 2017). ·OH-induced K^+^ efflux was much stronger in the elongation zone than in mature epidermis (Shabala *et al.*, 2016), and it was causally associated with cell fate determination under stress conditions (Demidchik *et al.*, 2010). A similar scenario may be envisaged for plants exposed to toxic metals such as Cu^2+^ or Fe^3+^, as interaction between these transition metals and H_2_O_2_ may lead to the formation of ·OH through the Fenton reaction in the cell walls (Demidchik, 2015), triggering K^+^ loss through GORK. Cd triggers the down-regulation of *GORK* transcripts in WT Arabidopsis plants, whereas no significant changes were observed in *RBOH C*, *D*, and *F* mutants (Hafsi *et al.*, 2022). Interestingly, the basal level of GORK transcripts in control conditions was reduced significantly in *AtrbohC*, and to a lesser extent in *AtrbohD* and *AtrbohF,* thus suggesting an RBOH-dependent regulation of GORK at the transcriptional level and possibly at the post-translational level (Hafsi *et al.*, 2022).

Tran *et al.* (2013) also reported that, in addition to a rapid activation of GORK at the single-channel level, ROS-dependent post-transcriptional regulation of GORK channels may occur. The abundance of GORK channel transcripts increases in a time-dependent manner after ozone (O_3_) treatment, and Tran *et al*. (2013) attributed this effect to pre-mRNA GORK splicing. It should also be noted that GORK transcript levels are increased in plants exposed to abiotic stresses, specifically salinity (Adem *et al.*, 2014).

Some reports on guard cells have shown that both inward- and outward-rectifying K^+^-selective channels in guard cells may be inhibited by H_2_O_2_ (e.g. in *V. faba*; Zhang *et al.*, 2001; Köhler *et al.*, 2003). At the same time, Laohavisit *et al.* (2012) have demonstrated the presence of an additional K^+^ efflux pathway that is catalyzed by annexins, as an Arabidopsis loss-of-function mutant for annexin1 (*Atann1*) lacked ·OH- activated Ca^2+^- and K^+^-permeable conductance in root epidermis. Thus, at least two mechanisms seem to coexist and act in concert to amplify ·OH-induced K^+^ efflux.

### Other ROS-regulated channels and transporters

Exogenous H_2_O_2_ stimulates anion efflux in cultured Arabidopsis cells (Trouverie *et al.*, 2008); however, this effect appears to be indirect and related to the activation of Ca^2+^ conductance, which in turn activates Cl^–^ currents (Demidchik, 2015). Kadono *et al.* (2010) demonstrated that 3 min of O_3_ exposure was enough to activate anion currents in cell suspensions and depolarize the plasma membrane. However, as the authors used a voltage-clamp approach on intact cells, it cannot be excluded that the reported effect was indirect and also mediated by O_3_-induced changes in cytosolic free Ca^2+^. More direct experiments on protoplasts are therefore needed.

Organelle-based ion channels also appear to be a target for ROS regulation. Pastore *et al*. (2007) showed that ROS can quickly stimulate the ATP-sensitive plant mitochondrial K^+^ channel (PmitoKATP) in wheat. The activity of mitochondrial K-ATP^+^ in pea was also modulated by NO and H_2_O (Chiandussi *et al.*, 2002), leading to a release of cytochrome *c* and consequent programmed cell death. Activation of tonoplast-based Ca^2+^-, K^+^-, and Na^+^-permeable SV (slow vacuolar) channels encoded by the *TPC1* gene by physiologically relevant concentrations of H_2_O_2_ has also been demonstrated in direct patch-clamp experiments (Pottosin *et al.*, 2009).

To the best of our knowledge, no direct reports of ROS-induced activation of members of the HAK/KUP family of high-affinity K^+^ transporters has been reported in plants, although many papers have reported an apparent correlation between changes in the expression levels of these transporters and ROS metabolism in plants. Overexpression of *MiHAK14* from *Mangifera indica* in Arabidopsis enhanced plant tolerance to K^+^ depletion and NaCl stress by improving ROS scavenging ability (Zhang *et al.*, 2022). Similar findings were reported for *Casuarina equisetifolia*, where *CeqHAK6* and *CeqHAK11* increased antioxidative defences (Wang *et al*., 2023). In turn, ROS accumulation may affect the transcript levels of *HAK*/*KUP* family genes. In Arabidopsis, plants overexpressing *RCI3*, a member of a family of peroxidases, showed higher ROS production and increased *AtHAK5* expression levels (Kim *et al*., 2010). *AtHAK5* and *AtKUP8* have been also reported to be regulated by RBOH-dependent H_2_O_2_ (Wang *et al.*, 2021; Hafsi *et al.*, 2022). We have also identified C^762^ from HAK5 as a target for *S*-nitrosylation (Supplementary Table S1). Arabidopsis *Atkup8-2* mutant plants showed lower accumulation of H_2_O_2_ compared with WT plants when grown in the presence of heavy metals (Sanz-Fernández *et al.*, 2021), and plants overexpressing *PvHAK16* from seashore paspalum (*Paspalum vaginatum*) showed increased accumulation of ROS under salt stress (Dai *et al.*, 2022). Members of the KUP family, such as KUP5 and KUP6 and the antiporter KEA1 for potassium transport, are differentially regulated in *nia1nia2noa1-2* triple mutants compared with the WT (Table 1), implying a role for NO in their operation.

Additionally, some proteins can change their functionality after oxidation. This is the case for the heme transporter HmuR in the bacterium *Burkholderia thailandensis T6SS4*. HmuR is a redox-regulated dual-functional transporter that under normal conditions transports heme iron but can transport zinc under oxidative stress, following the formation of an intramolecular disulfide bond in the protein (Si *et al.*, 2017).

## ROS- and NO-sensing mechanisms involved in the regulation of ion channels and transporters

One of the intriguing questions in plant redox biology is the identification of ROS/redox sensors (Sierla *et al.*, 2016). In general, ROS have been shown to change the activity of a large number of regulatory enzymes, such as various kinases (e.g. MAP and other Ser/Thr kinases) and phosphatases (Apel and Hirt, 2004; Van Breusegem *et al.*, 2008; Pitzschke and Hirt, 2009).

Owing to its low redox buffering capacity, the apoplast is an excellent medium for ROS signal propagation, and it harbors a large number of cysteine-rich kinases that could possibly participate in ROS-sensing mechanisms (Bourdais *et al.*, 2015). For example, cysteine-rich receptor-like kinases (CRKs) represent one of the largest subgroups of receptor-like kinases and are ideally suited for the role of ROS sensors (Wrzaczek *et al.*, 2010; Bourdais *et al.*, 2015). The CRKs possess two cysteine-rich DUF26 domains (C-X8-C-X2-C-motifs) and, upon ROS binding, could undergo redox modifications leading to conformational changes and downstream signaling. In stomata guard cells, apoplastic ROS signals may be perceived by GHR1 (GUARD CELL HYDROGENPEROXIDE-RESISTANT1), an atypical plasma membrane-associated leucine-rich repeat receptor-like kinase (Hua *et al.*, 2012). Another possible candidate sensor is CPK21 (Ueoka-Nakanishi *et al.*, 2013), which can also activate guard cell-expressed anion channels (Geiger *et al.*, 2010). Furthermore, the expression of *IRT1* in Arabidopsis mutants lacking MAPK3 and MAPK6 (*mpk3* and *mpk6*, respectively) was shown to be down-regulated under Fe deficiency (Ye *et al.*, 2015). MAPK3 and MAPK6 participate in a MAPK pathway downstream of ROS, contributing to both abiotic and biotic stress signaling (Opdenakker *et al.*, 2012; Jalmi *et al.*, 2018), suggesting the existence of H_2_O_2_-dependent regulation of *IRT1* via the MAPK pathway.

Plant transcription factors may also potentially assume the role of ROS sensors, as mentioned before (Hong *et al.*, 2013; Demidchik, 2015). Known examples include the transcription factor TGA1, which possesses two specific cysteine residues (C^260^ and C^266^) that could be oxidized (Després *et al*., 2003). Another example is the heat shock transcription factors, which could also be involved in direct ROS sensing (Hong *et al.*, 2013). *Brassica juncea* BjCdR15, a bZIP transcription factor orthologue of Arabidopsis TGA3, is a regulator of Cd uptake, translocation, and accumulation in shoots, and confers Cd tolerance in transgenic plants by regulating the expression of *AtHMA4*, *AtNRAMP3*, *AtABCC3*/*AtMRP3*, and *AtABCG36/AtPDR8* (Farinati *et al.*, 2010). *TGA3* transcript levels were up-regulated by Cd exposure in WT and *AtrbohC* plants, whereas no significant changes were observed in *AtrbohD* and *AtrbohF*, suggesting that H_2_O_2_ from RBOHD and RBOHF could regulate *TGA3* (Hafsi *et al.*, 2022). Herrera-Vásquez *et al.* (2021) have provided evidence to support the idea that TGA class II (TGA2/5/6) transcription factors represent a redox regulatory node in biotic and abiotic stress responses. Additionally, TGA2/5/6 impact on the cellular redox state by controlling the expression of genes responsible for restraining ROS accumulation (Herrera-Vásquez *et al.*, 2021).

Transcriptomic analyses of genes induced by increasing intracellular H_2_O_2_ levels in *cat2* Arabidopsis mutants and Arabidopsis mutants with altered ROS production have allowed the identification of several transcription factors in the WRKY, AP2/ERF, MYB, NAC, HSF, and ZAT families (He *et al.*, 2018; Terrón-Camero *et al*., 2022). WRKY46 plays an important role in the control of root-to-shoot Fe translocation under Fe deficiency conditions via the direct regulation of *VTL1* transcript levels (Yang *et al.*, 2016). It should be also borne in mind that ABC transporters are targets of nuclear factor *erythroid 2-related factor* 2 (*Nrf2*) in mice (Aleksunes and Klaassen, 2012) and *Nrf2* is activated by ROS in animal cells (Marinho *et al.*, 2014). Other transcription factors regulated by ROS in animal cells include CREB, TP53, NOTCH, NF-kB, SP1, HIF-1, SREBP-1, and HSF1, which have been considered as H_2_O_2_ sensors (Marinho *et al.*, 2014). Moreover, in plant cells, the transcription factor WRKY13 activates *AtABCG36* expression to positively regulate Cd tolerance (Sheng *et al.*, 2019; Li *et al.*, 2022). WRKY transcription factors have been identified as potential downstream targets of MAPKs (Jalmi *et al.*, 2018). The fact that ROS can lead to the activation of MAPK kinases (Li *et al.*, 2022; Torres and Forman, 2003) may suggest that H_2_O_2_ can regulate *ABCG36* expression via MAPK-dependent activation of *WRKY13*. The zinc finger transcription factor ZAT12 has been reported to be regulated at the transcriptional and post-translational levels by ROS in Arabidopsis, and to be up-regulated by prolonged Fe deficiency. Thus, ZAT12 could be involved in the cross-talk between ROS and Fe uptake regulation (Gratz *et al.*, 2021).

While the operation of the above sensory molecules is plausible and supported by numerous pieces of evidence, none of these models can explain the rapid activation of ion channels reported in patch experiments using a single-channel mode (e.g. Demidchik *et al.*, 2007), suggesting direct ROS sensing by ion channel(s) per se. Garcia-Mata *et al*. (2010) demonstrated that the SKOR K^+^ efflux channel was activated by H_2_O_2_ when heterologously expressed in HEK293 cells. Moreover, the substitution of the C^168^ residue on the S3 α-helix of the voltage sensor complex by another amino acid led to the loss of sensitivity of SKOR to H_2_O_2_ (Garcia-Mata *et al*., 2010). As the above cysteine residue also exists in GORK (Demidchik *et al.*, 2014), this could explain a direct activation of the GORK channels by ROS, discussed above.

As for the NSCC channels, the bioinformatics analysis revealed the presence of two candidates among the CNGCs, namely CNGC19 and CNGC20, that possess putative Cu/Fe-binding sites that could represent the cysteine metal pockets situated in the first cytosolic domain of CNGC (Demidchik *et al.*, 2014). Such cysteine residues have been shown to be responsible for **·**OH-mediated activation of Ca^2+^-permeable channels in animal cells (Simon *et al.*, 2004).

Regarding NO signaling, NRAMP3 and NRAMP4 have been reported recently to be transcriptionally regulated by *S*-nitrosylated bHLH29, bHLH38, and bHLH101 transcription factors (Shee *et al.*, 2022). An NO-sensing mechanism based on the N-degron pathway-mediated degradation of clade VII ethylene response factor (ERF) transcription factors (ERFVIIs) has been reported to control a wide array of plant developmental and stress-related responses (Gibbs *et al.*, 2014; Abbas *et al.*, 2015). The ERFVIIs group comprises three constitutively expressed genes, *RAP2.2*, *RAP2.3/EBP*, and *RAP2.12*, as well as two hypoxia-inducible genes, *HRE1* and *HRE2*, which have been demonstrated to be substrates of the E3 ubiquitin ligase PROTEOLYSIS6 (PRT6), a key regulator of the Cys/Arg branch of the N-degron pathway (Gibbs *et al.*, 2015). RAP2.3/EBP/ERF72 could directly bind to the promoter regions of Fe-deficiency response genes including the Fe transporter *IRT1*, *HA2*, and *CLH1* to exert negative regulation on responses to Fe deficiency (Liu *et al.*, 2017). In woody apple plants, MbERF72 suppress Fe uptake by modulating an H^+^-ATPase and, consequently, the rhizosphere pH (Zhang *et al.*, 2020). The expression of *RAP2.2* is controlled by two WRKY family transcription factors, WRKY33 and WRKY12, during hypoxia-triggered responses (Tang *et al.*, 2021). The WRKY33-ATL31-IRT1 module has been recently reported to play a crucial role in blocking Cd absorption to prevent metal toxicity in Arabidopsis (Zhang *et al.*, 2023). Extensive work will be needed to assess whether ERFVII signaling and WRKY-related regulation of metal homeostasis are linked and, if so, to what extent.

## Conclusions and perspectives

Plants have developed an evolutionary strategy to regulate their response to environmental changes, including nutritional imbalance and toxic heavy metals and salinity, through a site-specific ROS and NO footprint (Gibbs *et al*., 2014; Zhu *et al.*, 2020; Romero-Puertas *et al.*, 2022). Some of the targets of ROS- and NO-dependent transcriptional regulation are ion/metal transporters, with major implications for plants’ capacity to sense and adapt to biotic and abiotic stresses.H_2_O_2_ and NO modify reversibly specific cysteine residues (or tyrosine residues in the case of ONOO^–^) in proteins. ROS and NO interact both with each other and with proteins involved in their production and metabolism, regulating their own levels and the cellular redox equilibrium. The identification of redox-sensitive nutrient sensors in plant cells, their specificity, and the mechanisms involved in decoding these signals is one of the challenges for the field. A number of metal transporters, ion channels, MAPKs, and transcription factors appear to be regulated transcriptionally by ROS and NO, although the mechanisms underlying the network involved in this regulation is far from being completely known. Further methodological improvements will allow better identification of membrane proteins to more completely address the relevance of redox changes in the functionality of metal and ion transporters. However, the **·**OH-dependent activation of Ca^2+^-permeable NSCC and SKOR has been well established. Here, we have also implemented our knowledge of NO-dependent regulation of metal transporters by the identification of cysteine and tyrosine targets of NO by an *in silico* analysis, which requires confirmation by further proteomic approaches. Interestingly, some transporters are targets of both ROS- and NO-dependent PTMs, suggesting that these reactive species are an important hub in the regulation of their functionality. Additionally, the specific subcellular sites of production of ROS and NO could trigger specific patterns of PTM and signaling, which require further deep analysis. All this information could be of interest in designing new strategies to fortify crops, improving plant resilience against nutritional imbalance and salinity, and designing new phytoremediation methodologies based on redox biochemistry governed by ROS and NO.

## Supplementary data

The following supplementary data are available at *JXB* online.

Table S1. Prediction of *S*-nitrosylation and Y nitration sites of Arabidopsis metal transporters.

erad349_suppl_Supplementary_Tables_S1
