# Supplementary material for: Reactive oxygen species- and nitric oxide-dependent regulation of ion and metal homeostasis in plants
Source: J Exp Bot. 2023 Sep 5;74(19):5970–88. doi: 10.1093/jxb/erad349 (PMC10575707; doi:10.1093/jxb/erad349)
Supplement: erad349_suppl_Supplementary_Tables_S1 [file erad349_suppl_supplementary_tables_s1.pdf]

Supplementary table 1. Prediction of S-nitrosylation and Y nitration sites of Arabidopsis metal transporters.

	GPS-SNO (C-nitrosylation)					GPS-YNO2 (Y nitration)					DeepNitro							
											S-nitrosylation				Y nitration			
ID	Position	Peptide	Score	Cutoff	Cluster	Position	Peptide	Score	Cutoff	Cluster	Position	Peptide	Score	Cutoff	Position	Peptide	Score	Cutoff
>sp|Q9LVM1|ABCB25	405	LSTAMVLCSQGIMNG	935	443	Cluster B	130	ILRTLAGYLWMRDNP	1	828	Cluster C	639	KSPAILLCDEATSAL	0.401	0.37	360	KYFNNEGYEAEKYDQ	0.341	0.295
	639	KSPAILLCDEATSAL	451	443	Cluster B	707	LLGKSGRYAQLWTQQ	166	828	Cluster C								
	680	RLTTAMQCDEIVVLE	109	443	Cluster B													
>sp|Q0WML0|ABCB27						282	ESYMVSQYSKKVDET	798	102	Cluster E					448	ANLIERFYDPLKGKI	0.323	0.295
						342	ALTSFILYSLTVGSS	834	828	Cluster C								
						627	LLSLNGIYTNLVKRQ	699	828	Cluster C								
>sp|Q9C8G9|ABCC1	10	FEPLDWYCKPVPNGV	902	484	Cluster A	822	TVKEEGTYEELCHSG	1	828	Cluster C	1300	GRILIDECDIGRFGL	0.462	0.37				
						1091	STTRSPVYAQFGEAL	819	725	Cluster A	1423	IREEFKSCTMLIIAH	0.468	0.37				
						1110	SIRAYKAYDRMAEIN	829	828	Cluster C								
						1211	SVERVGNYIEIPSEA	307	65	Cluster B								
						1480	TGTANAEYLRSITLE	177	65	Cluster B								
						1608	NRMQHPDYNLEGKSF	145	828	Cluster C								
>sp|Q42093|ABCC2	10	FEFIEWYCKPVPNGV	279	484	Cluster A	822	TVKEEGTYEELSSNG	456	828	Cluster C	782	GQQVFEKCIKRELGQ	0.403	0.37				
											1305	GRILIDDCDVGKFGL	0.47	0.37				
											1428	IREEFKSCTMLIIAH	0.466	0.37				
>sp|Q9LK64|ABCC3						734	KPMERERYDKVLEAC	772	725	Cluster A	1291	LVLRGITCTFKGGLR	0.479	0.37	707	KVCGTKAYVAQSPWI	0.649	0.295
						1505	FSKLVAEYTSRSSSS	131	102	Cluster E								
>sp|Q8VZZ4|ABCC6	1365	VGQRQLVCLGRVLLK	457	443	Cluster B	14	SLLLQPIYLSVLSFF	228	65	Cluster B	663	ISGNLKVCGRKAYIA	0.409	0.37	668	KVCGRKAYIAQSPWI	0.639	0.295
						695	KPMEREWYDRVLEAC	784	725	Cluster A	835	ALATIDSCETGYASE	0.398	0.37	695	KPMEREWYDRVLEAC	0.348	0.295
											1241	MVLHGLTCTFPGGLK	0.37	0.37	839	IDSCETGYASEKSTT	0.32	0.295
											1257	GIVGRTGCGKSTLIQ	0.455	0.37	1110	IMRLSDCYSRLKFHS	0.505	0.295
>sp|Q9XIE2|ABCG36	384	TTFQIVKCLQQIVHL	304	443	Cluster B	249	VPRKTSAYISQNDLH	98	65	Cluster B	272	TLDFSARCQGVGTRY	0.385	0.37	320	KNSLVTDYTLKILGL	0.487	0.295
						933	AGRKTGGYIEGDVRI	465	65	Cluster B					933	AGRKTGGYIEGDVRI	0.586	0.295
															1100	KRGGQVIYAGPLGQN	0.493	0.295
>sp|Q9M9E1|ABCG40						642	VGRLFKQYILLVLMN	149	65	Cluster B					499	AALTTKKYGVGIKEL	0.424	0.295
						891	AGRKTGGYIDGNITI	572	65	Cluster B					891	AGRKTGGYIDGNITI	0.378	0.295
						1386	KQFIREFYGYREGFL	737	725	Cluster A								
>sp|Q9C9W0|ABCI17															140	KLSDEEVYKLLSLAD	0.321	0.295
>sp|Q39065|COPT1	170	VPPSGCAC*******	576	443	Cluster B													
>sp|Q9STG2|COPT2	156	LLPPSSGCVC*****	614	443	Cluster B													
	158	PPSSGCVC*******	859	443	Cluster B													
>sp|Q9FGU8|COPT3																		
>sp|Q8SAA5|COPT4																		
>sp|Q93VM8|COPT5																		
>sp|Q8GWP3|COPT6																		
>sp|Q9M3H5|HMA1	395	PFLSTAACRGSVYRA	565	443	Cluster B	587	DAVNASSYGKDFVHA	343	102	Cluster E	655	VGITEVYCNLKPEDK	0.452	0.37	532	IFVESFEYFPGRGLT	0.639	0.295
	490	ITCCIPNCEKEALAV	402	443	Cluster B													
>sp|Q9SZW4|HMA2	17	YFDVLGICCTSEVPL	322	484	Cluster A	10	SKKMTKSYFDVLGIC	42	725	Cluster A	235	GVVVDGNCEVDEKTL	0.443	0.37	10	SKKMTKSYFDVLGIC	0.515	0.295
	18	FDVLGICCTSEVPLI	902	484	Cluster A	179	WLQSRASYKASAVMQ	497	828	Cluster C	279	TTALAEDCVVAKMAK	0.376	0.37	378	LLIKGADYLETLAKI	0.331	0.295
	905	KVKSDSHCKSNCSSR	538	443	Cluster B						486	RIASRAGCLSVPDID	0.477	0.37	504	KGGKTIGYVYVGETL	0.628	0.295
											744	DKHCKPGCCGTKTQE	0.401	0.37	506	GKTIGYVYVGETLAG	0.316	0.295
>sp|P0CW77|HMA3A	23	YFDVVGICCSSEVSI	585	484	Cluster A	531	NLLDGCRYGVAQALK	399	828	Cluster C	241	GVVVDGSCDVDEKTL	0.421	0.37	16	KMNLQTSYFDVVGIC	0.626	0.295
	384	FLIKTGDCLETLAKI	717	443	Cluster B						285	TTALARDCVVAKMTK	0.47	0.37	512	KRGKTIGYIYMGAKL	0.64	0.295
											384	FLIKTGDCLETLAKI	0.456	0.37	514	GKTIGYIYMGAKLTG	0.478	0.295
											492	RIAQRAGCLTDNVPD	0.466	0.37				
											529	SFNLLDGCRYGVAQA	0.385	0.37				
>sp|P0CW78|HMA3B	23	YFDVVGICCSSEVSI	585	484	Cluster A						241	GVVVDGSCDVDEKTL	0.421	0.37	16	KMNLQTSYFDVVGIC	0.626	0.295
	384	FLIKTGDCLETLAKI	717	443	Cluster B						285	TTALARDCVVAKMTK	0.47	0.37	512	KRGKTIGYIYMGAKL	0.64	0.295
											384	FLIKTGDCLETLAKI	0.456	0.37	514	GKTIGYIYMGAKLTG	0.478	0.295
											492	RIAQRAGCLTDNVPD	0.466	0.37				
>sp|O64474|HMA4	27	YFDVLGICCTSEVPI	541	484	Cluster A	20	VKKLQKSYFDVLGIC	784	725	Cluster A	245	GIVVDGNCEVDEKTL	0.474	0.37	1150	GKSCCRSYAKELCSH	0.311	0.295
	315	SQRLIDKCSQYYTPA	94	484	Cluster A	189	WLETRASYKATSVMQ	497	828	Cluster C	289	TTSLAGDCVVAKMAK	0.405	0.37				
											496	KIASRAGCSTVPEIE	0.48	0.37				
											714	KKIGNKKCYRASTSK	0.448	0.37				
											865	QVESVGDCKSGHCEK	0.467	0.37				
											941	LIKDEGNCKSGSENM	0.376	0.37				
											1130	KVKIPEACASKCRDR	0.408	0.37				
>sp|Q9SH30|HMA5	10	TKLLSLTCIRKERFS	361	484	Cluster A	486	AKVASDGYVIWGQSH	47	65	Cluster B	10	TKLLSLTCIRKERFS	0.411	0.37	924	FSRIRLNYVWALGYN	0.389	0.295
	65	LGMTCSACAGSVEKA	875	443	Cluster B						726	NPAWPEACDFVSITG	0.47	0.37				

>sp|Q38856|IRT1	34 TSTAPEECGSESANP	837	443 Cluster B				337 LIAALLGCGGMSIIA	0.387	0.37			
	42 GSESANPCVNKAKAL	54	443 Cluster B									
	91 NIFTIIKCFASGIIL	462	443 Cluster B									
>sp|O81850|IRT2	332 SIKLQIKCFTAALLG	918	443 Cluster B									
>sp|Q8LE59|IRT3												
>sp|Q9S9N4|MRS2-1				132 LDNYVLRYVVELQQR	172	65 Cluster B				259 DGDMAEMYLTEKKRR	0.316	0.295
				175 FENSSPDYLPFEFRA	2	65 Cluster B						
				259 DGDMAEMYLTEKKRR	577	65 Cluster B						
				354 KLTSLKEYIDDTEDF	88	65 Cluster B						
>sp|Q9FLG2|MRS2-2				6 **MAQNGYLVPADPS	209	65 Cluster B				66 ILDPNLSYPSTILGR	0.639	0.295
				229 DDDMADLYLSRKLSS	791	65 Cluster B						
				250 SIGEPNWYTTSPTIG	218	828 Cluster C						
				305 RLTTLREYIDDTEDY	191	65 Cluster B						
				312 YIDDTEDYINIQLDN	386	65 Cluster B						
				388 IIMSYARYKGLVGS*	399	828 Cluster C						
>sp|Q9LJN2| MRS2-3	204 EFVALEACLEAASSS	668	443 Cluster B	277 DEDMAEMYLTEKLAQ	116	65 Cluster B				86 ILDPLLSYPSTVLGR	0.622	0.295
				393 KLSTLREYVDDTEDY	251	65 Cluster B						
>sp|Q93ZD7|MRS2-4				267 NEDMADLYLTRKWIQ	88	65 Cluster B	79 GAMEVVECDKSTIIK	0.443	0.37			
				355 YIDDTEDYVNIQLDN	33	65 Cluster B						
>sp|Q9ZPR4|MRS2-5	216 TQKVQKVCDEIEHLM	645	484 Cluster A	233 DDDMAEMYLTEKKER	688	65 Cluster B				90 LLDPLFIYPSSILGR	0.369	0.295
				333 KLSSLKEYIDDTEDL	27	65 Cluster B						
>sp|Q1PE39|MRS2-6							53 KKRRGGVCLWTRFDR	0.385	0.37			
>sp|Q304A0|MRS2-7				220 DGDMADLYLTRKLVG	549	65 Cluster B				68 ILDPNLFYPSAILGR	0.627	0.295
				297 KLTELREYIDDTEDY	274	65 Cluster B						
				304 YIDDTEDYINIQLDN	353	65 Cluster B						
>sp|P0CZ21|MRS2-8	144 EVALEAICSFLAART	596	484 Cluster A	212 DEDMAELYLSRKLAG	865	65 Cluster B				38 TVLDVDKYVIMHRVQ	0.477	0.295
				374 TIMSFARYKKLFGF*	78	828 Cluster C				60 ILDPNLFYPSAILGR	0.627	0.295
>sp|Q9LXD4|MRS2-9				299 KLAELREYLDDTEDY	112	65 Cluster B						
				306 YLDDTEDYINFQLAS	888	65 Cluster B						
				381 IIISYARYKKLVGN*	813	828 Cluster C						
>sp|Q9SAH0|MRS2-10				132 LDNYVLRYVVELQQR	177	65 Cluster B				272 KRMEGSLYGDQSLPV	0.32	0.295
				259 DGDMAEMYLTEKKKR	642	65 Cluster B						
				355 KLTSLKEYIDDTEDF	88	65 Cluster B						
>sp|Q058N4|MRS2-11	363 YLQRCESCHGQAERL	755	443 Cluster B									
>sp|Q9SAH8|NRAMP1	89 WIILVASCAALVIQS	554	443 Cluster B									
	418 PLLKFTSCKTKMGSH	755	443 Cluster B									
>sp|Q9C6B2|NRAMP2							393 RAVITRSCAIVPTMI	0.375	0.37			
>sp|Q9SNV9|NRAMP3				22 EEEEETAYDETEKVH	792	725 Cluster A						
>sp|Q9FN18|NRAMP4				18 LASEERAYEETEKVL	633	725 Cluster A						
>sp|Q9SN36|NRAMP5												
>sp|Q9S9N8|NRAMP6	81 WIILVASCAALVIQS	685	443 Cluster B									
>sp|O23482|OPT3				147 ITVMKAYYKQSLSFI	187	102 Cluster E						
>sp|Q9T095|OPT6												
>sp|O82485|OPT7				22 KDLTNPSYASSSSSS	73	725 Cluster A						
				765 IVEGCPLYT******	617	828 Cluster C						
>sp|O22397|POT1	376 SQCCALDCFPRVKII	592	443 Cluster B	573 VKSVQVPYVGEEERF	298	65 Cluster B				394 SKIHGQIYIPEVNWM	0.636	0.295
	644 VRRKKEECMEIMEAK	739	443 Cluster B	657 AKEAGVAYILGHSYA	567	65 Cluster B						
				711 LEVGMVYYV******	91	51 Cluster D						
>sp|O22881|POT2	9 DLNLGKCCGSRSSKK	984	484 Cluster A				594 VDHRSYRCIVRYGYR	0.441	0.37	393 DKMHGQIYIPEINWM	0.437	0.295
	138 PEKNHDSCVKRYLEK	929	443 Cluster B				771 YNFLRRNCRGPDVAL	0.428	0.37	764 RLAVNFGYNFLRRNC	0.4	0.295
	435 AVMLVTTCLTSLVIV	364	443 Cluster B							792 LLEVGMVYVV*****	0.513	0.295
>sp|Q9FE38|POT3	8 MADRRNRCNQILLLA	22	443 Cluster B	676 LLKLRAEYEQELPRL	697	102 Cluster E	581 KTYRMYRCIIRAGYK	0.478	0.37	720 AKDAEVAYIVGHGHV	0.559	0.295
	752 YSFLRKNCRSPGVML	563	484 Cluster A	774 IKVGMNYYL******	67	65 Cluster B						
>sp|Q9LD18|POT4				692 RSVYEDEYPQGQVRR	873	725 Cluster A	586 RFLIGRVCPKPYRMY	0.464		128 ADEELSAYKFGPSTD	0.32	0.295
				734 AKEAGVAYIMGHSYV	498	65 Cluster B	595 KPYRMYRCIVRYGYK	0.471		394 KHIYGQIYIPEINWI	0.507	0.295
				788 IEVGMIYYV******	736	51 Cluster D				740 AYIMGHSYVKSRKSS	0.373	0.295
										759 KMAIDIGYSFLRKNC	0.398	0.295
>sp|Q9M7K4|POT5	762 YNFLKKNCREGDKAL	743	484 Cluster A	507 YKFTSGGYLPLTITV	116	65 Cluster B	762 YNFLKKNCREGDKAL	0.475	0.37	755 KFIVNHAYNFLKKNC	0.359	0.295
				684 TVVPSSNYVPSSGRI	121	65 Cluster B				783 LLKVGMTYEL*****	0.439	0.295
				730 AREKGMVYLMGETEI	228	65 Cluster B						
				783 LLKVGMTYEL*****	428	51 Cluster D						
>sp|Q8W4I4|POT6	234 VILLWLMCISAIGVY	755	443 Cluster B	8 MEIESGSYQNAKKES	185	725 Cluster A	598 KEFRIYRCIVRFGYR	0.464	0.37	397 SKIHGQIYIPEINWI	0.375	0.295
	379 KQCSALGCFPKVKIV	571	443 Cluster B	780 TLEVGMIYNV*****	95	51 Cluster D	620 EFEGDLVCSIAEFIR	0.385	0.37	733 AYIMGNAYMKAKPGS	0.402	0.295

<table>
<tr><td></td><td></td><td></td><td></td><td></td><td></td><td></td><td></td><td></td><td></td><td>752 RLAINIGYEFLRRNT</td><td>0.52</td><td>0.295</td></tr>
<tr><td>>sp|Q9FY75|POT7</td><td>445 MTTATFSCIKQSTAL</td><td>65</td><td>443 Cluster B</td><td>856 LMQVGMTYMV*****</td><td>312</td><td>65 Cluster B</td><td>454 KQSTALGCFPRLKII</td><td>0.401</td><td>0.37</td><td>614 APGIGLLYNELVKGV</td><td>0.453</td><td>0.295</td></tr>
<tr><td></td><td></td><td></td><td></td><td></td><td></td><td></td><td>600 DLMRELGCNLGTIRA</td><td>0.452</td><td>0.37</td><td></td><td></td><td></td></tr>
<tr><td></td><td></td><td></td><td></td><td></td><td></td><td></td><td>664 RFLFRRVCTKSYHLF</td><td>0.409</td><td>0.37</td><td></td><td></td><td></td></tr>
<tr><td></td><td></td><td></td><td></td><td></td><td></td><td></td><td>673 KSYHLFRCIARYGYK</td><td>0.467</td><td>0.37</td><td></td><td></td><td></td></tr>
<tr><td>>sp|Q9M7J9|POT8</td><td>234 IVLAWLLCISTIGVY</td><td>63</td><td>443 Cluster B</td><td>196 MSKQQHQYVEVPVVC</td><td>7</td><td>65 Cluster B</td><td>600 KEYRLYRCIARYGYR</td><td>0.468</td><td>0.37</td><td>751 KIAINFGYDFLRRNS</td><td>0.484</td><td>0.295</td></tr>
<tr><td>>sp|O49423|POT9</td><td></td><td></td><td></td><td>806 LNVGQVFYV******</td><td>781</td><td>51 Cluster D</td><td>405 KQAVAHGCFPRVKIV</td><td>0.444</td><td>0.37</td><td>132 QGGTLAIYSLLCRHA</td><td>0.454</td><td>0.295</td></tr>
<tr><td></td><td></td><td></td><td></td><td></td><td></td><td></td><td>624 KTFRMFRCVARYGYK</td><td>0.475</td><td>0.37</td><td>157 SDEDLTTYSRTVSAE</td><td>0.547</td><td>0.295</td></tr>
<tr><td></td><td></td><td></td><td></td><td></td><td></td><td></td><td></td><td></td><td></td><td>394 QATISGTYSIVKQAV</td><td>0.322</td><td>0.295</td></tr>
<tr><td></td><td></td><td></td><td></td><td></td><td></td><td></td><td></td><td></td><td></td><td>423 KKFLGQIYCPDINWI</td><td>0.303</td><td>0.295</td></tr>
<tr><td></td><td></td><td></td><td></td><td></td><td></td><td></td><td></td><td></td><td></td><td>775 PKKIAIDYVYAFLAK</td><td>0.556</td><td>0.295</td></tr>
<tr><td>>sp|Q9SA05|POT10</td><td></td><td></td><td></td><td>795 LNVGQIFYV******</td><td>97</td><td>51 Cluster D</td><td>404 KQALAHGCFPRVKVV</td><td>0.425</td><td>0.37</td><td>156 TDEELTTYSRTTFHE</td><td>0.354</td><td>0.295</td></tr>
<tr><td>>sp|O64769|POT11</td><td></td><td></td><td></td><td>791 LNVGQIFYV******</td><td>97</td><td>51 Cluster D</td><td>405 KQALAHGCFPRVKVV</td><td>0.452</td><td>0.37</td><td>405 KQALAHGCFPRVKVV</td><td>0.452</td><td>0.37</td></tr>
<tr><td></td><td></td><td></td><td></td><td></td><td></td><td></td><td>624 KNFHMFRCVARYGYR</td><td>0.476</td><td>0.37</td><td>624 KNFHMFRCVARYGYR</td><td>0.476</td><td>0.37</td></tr>
<tr><td>>sp|O80739|POT12</td><td>429 MISATFSCVKQAMAL</td><td>63</td><td>443 Cluster B</td><td>714 VSVASDTYTDDLMAP</td><td>106</td><td>102 Cluster E</td><td>438 KQAMALGCFPRLKII</td><td>0.469</td><td>0.37</td><td>164 EGGTFALYSLICRYA</td><td>0.316</td><td>0.295</td></tr>
<tr><td></td><td></td><td></td><td></td><td>825 ILQAGMTYMV*****</td><td>33</td><td>65 Cluster B</td><td>657 KDYHMFRCIARYGYK</td><td>0.465</td><td>0.37</td><td>456 KKRIGQIYIPVINWF</td><td>0.405</td><td>0.295</td></tr>
<tr><td></td><td></td><td></td><td></td><td></td><td></td><td></td><td></td><td></td><td></td><td>772 ATDSGLTYLLAHGDV</td><td>0.617</td><td>0.295</td></tr>
<tr><td></td><td></td><td></td><td></td><td></td><td></td><td></td><td></td><td></td><td></td><td>795 VKKLVINYFYAFLRR</td><td>0.424</td><td>0.295</td></tr>
<tr><td>>sp|Q8LPL8|POT13</td><td>446 MTTATFTCIKQSIAL</td><td>728</td><td>443 Cluster B</td><td>823 LKKLVINYLYAFLRK</td><td>335</td><td>65 Cluster B</td><td>313 ALLLWFFCLAGIGIY</td><td>0.387</td><td>0.37</td><td>473 KKFIGQIYIPVLNWS</td><td>0.35</td><td>0.295</td></tr>
<tr><td></td><td></td><td></td><td></td><td>853 LMQVGMTYMV*****</td><td>321</td><td>65 Cluster B</td><td>455 KQSIALGCFPRLKII</td><td>0.456</td><td>0.37</td><td></td><td></td><td></td></tr>
<tr><td></td><td></td><td></td><td></td><td></td><td></td><td></td><td>674 RSYHLFRCVARYGYK</td><td>0.466</td><td>0.37</td><td></td><td></td><td></td></tr>
<tr><td>>sp|Q6R3L0|YSL1</td><td>615 AYFAIDMCVGTLIVF</td><td>484</td><td>443 Cluster B</td><td></td><td></td><td></td><td></td><td></td><td></td><td></td><td></td><td></td></tr>
<tr><td>>sp|Q6R3K9|YSL2</td><td>50 GIVYSVICLKLNLTT</td><td>462</td><td>443 Cluster B</td><td></td><td></td><td></td><td></td><td></td><td></td><td></td><td></td><td></td></tr>
<tr><td></td><td>605 GSFAIDMCIGSLVVY</td><td>913</td><td>443 Cluster B</td><td></td><td></td><td></td><td></td><td></td><td></td><td></td><td></td><td></td></tr>
<tr><td>>sp|Q2EF88|YSL3</td><td>613 GYFAIDMCVGSLIVF</td><td>793</td><td>443 Cluster B</td><td></td><td></td><td></td><td></td><td></td><td></td><td></td><td></td><td></td></tr>
<tr><td>>sp|Q6R3K8|YSL4</td><td></td><td></td><td></td><td>27 TNLDHGEYVPEWKEQ</td><td>121</td><td>65 Cluster B</td><td></td><td></td><td></td><td>446 DMSMPSTYGKTGLFI</td><td>0.618</td><td>0.295</td></tr>
<tr><td></td><td></td><td></td><td></td><td>492 MQDFKTGYLTLSSAK</td><td>2</td><td>65 Cluster B</td><td></td><td></td><td></td><td></td><td></td><td></td></tr>
<tr><td>>sp|Q9LUN2|YSL5</td><td>642 PYFAIDMCVGSLILF</td><td>538</td><td>443 Cluster B</td><td></td><td></td><td></td><td></td><td></td><td></td><td></td><td></td><td></td></tr>
<tr><td>>sp|Q6R3K6|YSL6</td><td></td><td></td><td></td><td>495 MQDFKTGYLTLSSAK</td><td>2</td><td>65 Cluster B</td><td></td><td></td><td></td><td></td><td></td><td></td></tr>
<tr><td>>sp|Q9SHY2|YSL7</td><td>617 GYFTIDMCLGSLILF</td><td>75</td><td>443 Cluster B</td><td></td><td></td><td></td><td></td><td></td><td></td><td>256 PTFGLKAYENKFYFD</td><td>0.305</td><td>0.295</td></tr>
<tr><td>>sp|Q6R3K4|YSL8</td><td></td><td></td><td></td><td></td><td></td><td></td><td></td><td></td><td></td><td></td><td></td><td></td></tr>
<tr><td>>sp|O81123|ZIP1</td><td>6 **MSECGCFSATTML</td><td>301</td><td>484 Cluster A</td><td></td><td></td><td></td><td></td><td></td><td></td><td></td><td></td><td></td></tr>
<tr><td>>sp|Q9LTH9|ZIP2</td><td>21 FLSIIFLCFSLILAH</td><td>418</td><td>443 Cluster B</td><td></td><td></td><td></td><td></td><td></td><td></td><td></td><td></td><td></td></tr>
<tr><td>>sp|Q9SLG3|ZIP3</td><td></td><td></td><td></td><td></td><td></td><td></td><td></td><td></td><td></td><td></td><td></td><td></td></tr>
<tr><td>>sp|O04089|ZIP4</td><td>10 SSTTKILCDAGESDL</td><td>563</td><td>484 Cluster A</td><td></td><td></td><td></td><td></td><td></td><td></td><td></td><td></td><td></td></tr>
<tr><td>>sp|O23039|ZIP5</td><td></td><td></td><td></td><td></td><td></td><td></td><td></td><td></td><td></td><td></td><td></td><td></td></tr>
<tr><td>>sp|O64738|ZIP6</td><td>4 ****MASCVTGTEAA</td><td>147</td><td>443 Cluster B</td><td></td><td></td><td></td><td></td><td></td><td></td><td></td><td></td><td></td></tr>
<tr><td></td><td>17 AAIRAAACRDGEEAS</td><td>694</td><td>484 Cluster A</td><td></td><td></td><td></td><td></td><td></td><td></td><td></td><td></td><td></td></tr>
<tr><td>>sp|Q8W246|ZIP7</td><td>7 *MAYSKACYKLTTIT</td><td>279</td><td>484 Cluster A</td><td>8 MAYSKACYKLTTITI</td><td>41</td><td>828 Cluster C</td><td>46 AESGDLSCHNNKEAQ</td><td>0.384</td><td>0.37</td><td></td><td></td><td></td></tr>
<tr><td></td><td></td><td></td><td></td><td>191 TLENGSSYVEKQEKV</td><td>251</td><td>65 Cluster B</td><td></td><td></td><td></td><td></td><td></td><td></td></tr>
<tr><td>>sp|Q8S3W4|ZIP8</td><td>41 ETDSTDSCIDKTKAL</td><td>723</td><td>443 Cluster B</td><td></td><td></td><td></td><td>41 ETDSTDSCIDKTKAL</td><td>0.4</td><td>0.37</td><td></td><td></td><td></td></tr>
<tr><td></td><td>184 TKERSSTCSKQLLRY</td><td>22</td><td>443 Cluster B</td><td></td><td></td><td></td><td></td><td></td><td></td><td></td><td></td><td></td></tr>
<tr><td></td><td>329 SVKLQLNCFGAALLG</td><td>185</td><td>443 Cluster B</td><td></td><td></td><td></td><td></td><td></td><td></td><td></td><td></td><td></td></tr>
<tr><td>>sp|O82643|ZIP9</td><td></td><td></td><td></td><td></td><td></td><td></td><td></td><td></td><td></td><td></td><td></td><td></td></tr>
<tr><td>>sp|Q8W245|ZIP10</td><td></td><td></td><td></td><td>202 NDKELGSYLQLLRYR</td><td>237</td><td>65 Cluster B</td><td></td><td></td><td></td><td></td><td></td><td></td></tr>
<tr><td>>sp|Q94EG9|ZIP11</td><td>128 PFAYMLACAGFMLTM</td><td>837</td><td>443 Cluster B</td><td></td><td></td><td></td><td></td><td></td><td></td><td></td><td></td><td></td></tr>
<tr><td></td><td>182 ILLIVALCFHSVFEG</td><td>65</td><td>443 Cluster B</td><td></td><td></td><td></td><td></td><td></td><td></td><td></td><td></td><td></td></tr>
<tr><td>>sp|Q9FIS2|ZIP12</td><td></td><td></td><td></td><td></td><td></td><td></td><td></td><td></td><td></td><td></td><td></td><td></td></tr>
</table>
